# Endocine™, N3OA and N3OASq; Three Mucosal Adjuvants That Enhance the Immune Response to Nasal Influenza Vaccination

**DOI:** 10.1371/journal.pone.0070527

**Published:** 2013-08-08

**Authors:** Tina Falkeborn, Andreas Bråve, Marie Larsson, Britt Åkerlind, Ulf Schröder, Jorma Hinkula

**Affiliations:** 1 Division of Molecular Virology, Department of Clinical and Experimental Medicine, Linköping University, Linköping, Sweden; 2 Swedish Institute for Communicable Disease Control (SMI), Stockholm, Sweden; 3 Eurocine Vaccines AB, Karolinska Science Park, Solna, Sweden; Osaka University, Japan

## Abstract

Annual outbreaks of seasonal influenza are controlled or prevented through vaccination in many countries. The seasonal vaccines used are either inactivated, currently administered parenterally, or live-attenuated given intranasally. In this study three mucosal adjuvants were examined for the influence on the humoral (mucosal and systemic) and cellular influenza A-specific immune responses induced by a nasally administered vaccine. We investigated in detail how the anionic Endocine™ and the cationic adjuvants N3OA and N3OASq mixed with a split inactivated influenza vaccine induced influenza A-specific immune responses as compared to the vaccine alone after intranasal immunization. The study showed that nasal administration of a split virus vaccine together with Endocine™ or N3OA induced significantly higher humoral and cell-mediated immune responses than the non-adjuvanted vaccine. N3OASq only significantly increased the cell-mediated immune response. Furthermore, nasal administration of the influenza vaccine in combination with any of the adjuvants; Endocine™, N3OA or N3OASq, significantly enhanced the mucosal immunity against influenza HA protein. Thus the addition of these mucosal adjuvants leads to enhanced immunity in the most relevant tissues, the upper respiratory tract and the systemic circulation. Nasal influenza vaccination with an inactivated split vaccine can therefore provide an important mucosal immune response, which is often low or absent after traditional parenteral vaccination.

## Introduction

Seasonal influenza infections lead to severe morbidity and mortality in sensitive individuals, including small children and individuals with heart and respiratory disorders [1]. Approximately 250 000–500 000 people either die directly from influenza or from secondary infections acquired following influenza infection every year [2]. The most important role of vaccination is to prevent hospitalization and mortality [3]. All inactivated influenza vaccines are currently administered with a syringe and needle, and poor immune responses and subsequent poor protection against disease, are often obtained when less immunogenic influenza vaccine strains are used [4]. It would be valuable to develop inactivated influenza vaccines that can be administered without injection and that also contribute to the induction of potent first-line defense, i.e., mucosal immunity [5], [6].

The best method for inducing local mucosal immunity is delivering the vaccine directly onto the mucosal surface where the microorganisms normally enter the body [7], [8], [9]. In the case of the influenza virus, this can be achieved by nasal or oral administration of the vaccine antigen. Nasally administered influenza vaccines often induce local mucosal influenza-specific IgA (secretory IgA). Secretory-IgA has been shown to have cross reactive properties [10], [11], so nasal vaccination inducing secretory-IgA may contribute to a broader immune response. A recent study confirmed that nasal IgA contributes to the efficacy of a nasally administered influenza vaccine [12]. These promising protective effects have been reported by others, such as nasal administration of the virus-like particle (VLP) H1N1 influenza vaccine that showed broad protective immunity in mice and ferrets against distant influenza strains [13], against which a non-adjuvanted parenteral vaccine failed to induce an immune response.

This study examined how three mucosal adjuvants influenced the humoral (local and systemic) and cellular influenza A-specific immune responses induced by the vaccine. We investigated in detail how the anionic Endocine™ and the cationic adjuvants N3OA and N3OASq mixed with a split inactivated influenza vaccine induced influenza A-specific immune responses compared to vaccine alone after intranasal immunization. The N3OASq is a cationic N3 adjuvant combined with squalene, and is a novel adjuvant combination presented for the first time in this manuscript. The study showed that a nasal split virus vaccine together with Endocine™ or N3OA induced significantly higher humoral and cell-mediated immune responses compared to the non-adjuvanted group. N3OASq only significantly increased the cell-mediated immune response. Furthermore, nasal administration of the influenza vaccine in combination with any of the adjuvants; Endocine™, N3OA or N3OASq significantly enhanced the mucosal immunity against influenza hemagglutinin (HA) protein.

## Materials and Methods

### 2.1. Mice

Eight to ten-week-old female BALB/c mice were purchased from Scanbur BK, Sollentuna, Sweden. All animal experiments in this study were approved by the local ethical committee at the Karolinska Institute.

### 2.2. Vaccination

Four groups of eight mice per group were vaccinated with split influenza antigen with or without adjuvant. The split influenza antigen was a commercially available trivalent split vaccine (Vaxigrip, LotNo 9627–1, Sanofi Pasteur, Bryssel, Belgium), containing A/Brisbane/59/2007 (H1N1), A/Brisbane/10/2007 (H3N2) and B/Brisbane/60/2008. The vaccine was concentrated 10 times using Amicon 10 K concentrator filters (Amicon, US). Mice were given 5 µL of vaccine in each nostril, corresponding to a total of 1.5 µg HA and 0–2% adjuvant. During vaccination, the mice were anaesthetized with isoflurane (Baxter International Inc, Deerfield, US). The four groups were immunized three times at three-week intervals (day 0, 21 and 42) with blood samples taken after the first and second immunization and the mice were sacrificed one week after the last immunization. One mouse in the N3OA group had to be removed earlier, due to health problems. After sacrifice the spleens were removed and splenocytes prepared for analysis [14]. Blood samples (serum) were taken from the tail vein after each vaccination and at the time of sacrifice. The blood samples were centrifuged and stored at −20°C. Nasal washes were also performed at sacrifice [15] and samples stored at −20°C for further analysis.

### 2.3. Adjuvants

The adjuvants used in this study have previously been described [16], [17], [18], [19]. The three different adjuvants were: the anionic Endocine™ (previously known as L3B), which consists of the endogenous lipids mono-olein and oleic acid; the cationic N3OA, which consists of oleylamine; and the cationic N3OASq, which consists of oleylamine and squalene (all from Eurocine Vaccine AB). The final concentrations for Endocine™, N3OA, and N3OASq when mixed with the influenza A vaccine were 2% for Endocine™, and 1% for the adjuvants N3OA and N3OASq.

### 2.4. Antibody measurement

#### 2.4.1. Enzyme-linked immunosorbent assay (ELISA)

Serum samples were analyzed for specific IgG, IgA, and subclass IgG (IgG1, IgG2a, IgG2b and IgG3) against recombinant HA (rHA) from A/Brisbane/59/07 (H1N1) and A/California/04/2009 (H1N1) (Protein Sciences corporation, Meriden, NJ) with an enzyme-linked immunosorbent assay (ELISA). ELISA was also used to analyze serum IgG and IgA against rHA from A/Brisbane/08 (H3N2) and B/Brisbane/60/08 (Protein Sciences). Serum IgA was also tested against two different vaccines; Vaxigrip (A/Brisbane/59/2007 (H1N1), A/Brisbane/10/2007 (H3N2) and B/Brisbane/60/2008) from season 2009/2010 and vaccine against influenza (A/California/7/2009 (H1N1), A/Perth/16/2009 H3N2 and B/Brisbane/60/2008) from season 2010/2011. Nasal washes were analyzed for mucosal IgA against rHA from A/Brisbane (H1N1). All sera and nasal washes from the sacrificed mice were individually analyzed. Serological responses were measured as previously described [19]. In brief, 96-well plates (NuncImmuno Plate, Polysorp, Roskilde, Denmark) were used for ELISA. Plates were coated with rHA (Brisbane or California at a concentration of 1–2 µg/mL or with vaccine at a concentration of 0.5 µg/mL. Sera were diluted ten-fold from a starting dilution of 1∶100 in ELISA-buffer (2.5% dry milk and 0.05% Tween^®^-20 (Sigma-Aldrich, S:t Louis, Mo) in PBS (PAA, Pasching, Austria)). For IgG measurement, goat-anti-mouse IgG (H+L)-HRP conjugate (Bio-Rad, Sundbyberg, Sweden) diluted 1∶3000 was used. IgA and IgG subclasses were measured with a mouse monoclonal antibody isotyping reagent (Sigma-Aldrich) according to the manufacturer's protocol in conjunction with peroxidase-conjugated anti-Goat IgG (Sigma-Aldrich) diluted 1∶20 000. For developing the reaction, O-phenylenediaminedihydrochloride (OPD) (Sigma-Aldrich) was used according to the manufacturer's protocol. Based on earlier studies, an OD of 0.2 was set as the cut-off value for positive samples. Calculation of the ratio between the IgG1 and IgG2a subclasses was also performed.

The strength of IgG binding to the HA of the Brisbane strain used in ELISA was assessed using the Urea-wash analysis (Avidity assay) as previously described [20], [21]. In brief, the same ELISA as above was used, but after the initial antibody-antigen incubation, the plates were washed with 8 M urea, pH 8 or wash buffer. Thereafter, the same conjugate and developer were used for the final step of the assay. An avidity index (AI) was then calculated by dividing the OD-value from the 8 M urea-washed wells with the OD value from the wash buffer-washed wells.

Mucosal wash IgA analyses (both total and influenza specific-IgA) were performed as previously described [18], [19]. Captive/IgA/IgE reagents (Biotech-IgG, Copenhagen, Denmark) were used as recommended by the manufacturer. The reagents were used to isolate and analyse HA-specific IgA from the nasal washings. 96-well plates were coated with rHA from A/Brisbane/59/2007 (H1N1) as mentioned previously. Total IgA quantities were determined using an in-house murine IgA capture ELISA, and commercial murine IgA (1 mg/mL, Sigma-Aldrich) was used for preparing a standard curve. Briefly, the purified IgA and the standard IgA were diluted in ten-fold serial dilutions. From each dilution, 100 µL was added to each well of a 96-microwell plate pre-coated with rabbit anti-murine IgA (Dakopatts AB, Sollentuna, Sweden). HRP-conjugated goat anti-murine IgA was used and the presence of bound conjugate was detected and measured as above. The total amounts of IgA in the mouse samples were determined by comparing the OD values of the test samples with the IgA standard. The influenza specific IgA was divided with the total IgA to get: influenza HA-specific IgA/mg total IgA.

#### 2.4.2. Hemagglutination inhibition (HAI)

HAI was performed as previously described [19] and according to the standard procedure [22]. In brief, sera were first RDE treated (RDE 11, Denka Seiken CO, Japan) to inactivate non-specific inhibitors. The initial dilution of the RDE-treated sera was 1:10. Influenza A/H1N1/Brisbane/2007 (Karolinska Institute, Stockholm, Sweden) was used in this assay. Red blood cells from chicken (Håtuna lab, Uppsala, Sweden) were used at a concentration of 0.5%. Mouse sera were analyzed individually after sacrifice. The HAI titer was defined as the highest dilution of the sera that was able to completely inhibit hemagglutination.

#### 2.4.3. Neutralization assay (NT-assay)

The neutralization assay (NT-assay) was performed as previously described [22], [23], with some modifications. In brief, Madin-Darby Canine kidney (MDCK) cells were grown to a confluent monolayer in a F96-well microtiter plate (Nunc Immuno Plate) in media (GIBCO^®^ RPMI 1640 with L-glutamine (Invitrogen, N.Y, USA)), 1% PEST, 8% FCS, 4 mM Na-pyruvate and 50 µM 2-Mercaptoethanol) in a 37°C humidified incubator with 5% CO_2_. The serum samples were diluted 1∶5 in serum-free media and inactivated at 56°C for 30 minutes before use. Serially diluted sera and 10 TCID_50_/well of A/H1N1/Brisbane virus (Karolinska Institute) were incubated together with 5 µg/mL trypsin for 1 h in a 37°C humidified incubator with 5% CO_2_, after which they were added to MDCK cells. After 2 h of incubation, the virus/sera mix was removed and 200 µL serum-free media with 5 µg/mL trypsin was added to each well. After four days of incubation in a 37°C humidified incubator with 5% CO_2_, the plates were fixed with acetone and analyzed with ELISA. A human IgG antibody diluted 1∶5000 was used together with a polyclonal Rabbit anti-human IgG/HRP (Dako, Glostrup, Denmark) diluted 1∶10 000. The plate was developed as described above.

### 2.5. Measurement of cytokines

#### 2.5.1. ELISpot

The cell-mediated immune responses were measured by ELISpot assays as described previously [24], [25], [26]. In brief, splenocytes from immunized mice were analyzed for influenza-specific cytokine secretion. For detecting interleukin-2 (IL-2) and interferon-gamma (IFN-γ), 96-well ELISpot plates were used according to the manufacturer's protocol (Mabtech, Nacka, Sweden). In short, the plates were activated with ethanol and coated with 100 µL/well of the capture antibody 1-D1K (15 µg/mL) or IL2-i/249 (15 µg/mL) overnight at 4°C. Then, 250 000 splenocytes were added to each well, either with or without stimulatory agents. The antigens used as stimulatory agents in the ELISpot assays were whole virus of A/H1N1/Brisbane/59/07 (Swedish Institute for Communicable Disease control (SMI), Sweden) whole virus of A/H1N1/California/04/2009 (SMI) and nucleo-protein (NP) peptide mix (SNLNDATYQRTRALV_141–155_, TYQRTRALV_147–155_ and TRALVRTGMDPRMCS_151–165_) from A/Brisbane (H1N1) (GeneScript, NJ, USA), the positive control provided in the kit and RPMI 1640-medium as a negative control. The plates were incubated in a 37°C humidified incubator with 5% CO_2_ overnight. The next day, biotinylated detection antibodies were added followed by streptavidin-ALP. The plates were developed with a substrate solution until spots became visible, and the color reaction was stopped by washing the plate extensively in tap water. The plates were air-dried and then read in an ELISpot reader (AID, Mannheim, Germany) to evaluate the result. Magnetic beads (microbeads specific for mouse CD4 (L3T4)) were used to deplete CD4+ T cells according to the protocol provided by the manufacturer (Miltenyi Biotec, Fisher Scientific, GTF, Gothenburg, Sweden).

### 2.6. Statistical analysis

Statistical analysis was performed using GraphPad Prism 5 (La Jolla, CA, US). To compare ELISA titer, HAI titers, NT-titers and ELISpot data between the four groups, a Kruskal-Wallis one way ANOVA was used to test if there was a significant difference between groups. The p-value of the ANOVA test was below 0.002 for all data sets. To check for significant difference between any two groups the Mann Whitney U test with Bonferroni correction was used. A p-value of <0.0017 was considered to be statistically significant. In ELISA and the NT-test the mice that responded with titers less than 100 was given a fictive value of 20 and in the HAI test, mice responding with titers less than 10 was given the value 5.

## Results

### 3.1. Serological responses

**Figure 1:**
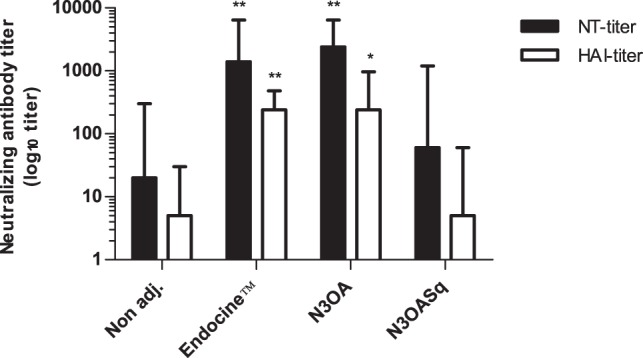
HAI and NT-antibody titers against influenza A/H1N1/Brisbane in serum after the final immunization. Mice were immunized with a split influenza vaccine (Vaxigrip) containing the A/H1N1/Brisbane/2007 strain with or without adjuvant. The groups were immunized three times with three-week intervals. The HAI and NT-antibody reactivity against influenza A/H1N1/Brisbane in serum after the final immunization are shown. Median and range is shown for each group. Values <10 in HAI was set as 5 and values <100 in the NT-assay was set as 20. Statistical significances compared to the non-adjuvanted group are indicated, *p<0.017 and **p<0.003.

The serum antibody responses induced by the unadjuvanted vaccine and the vaccine in combination with each of the mucosal adjuvants were compared by HAI. The mice received three nasal immunizations at three-week intervals. The two adjuvants Endocine™ and N3OA, significantly (p = 0.0005 and p = 0.0038, respectively) increased the HAI responses against A/Brisbane/2007 (H1N1) compared to the non-adjuvanted group (Fig. 1). In the Endocine™ group, 8 out of 8 mice responded with a median titer of 240 (min 120, max 480) and in the N3OA group 86% of the mice, responded with a median titer of 240 (min <10, max 960) against Brisbane. The HAI responses were poor in the two other groups. In the non-adjuvanted group, only one mouse responded with a titer of 30 and in the N3OASq group, two mice responded with the titer 60. In the NT-assay it was shown that NT-antibodies against A/Brisbane/2007 (H1N1) were induced in all mice in two of the groups; Endocine™ with a median titer of 1400 (min 400, max 6400) and N3OA with a median titer of 2400 (min 100, max 6400). The antibody titers in these two groups were significantly (p = 0.0008 and p = 0.0031 respectively) higher than those in the non adjuvanted group 1 (Fig. 1). In the N3OASq group, 50% of the mice produced NT-antibodies against Brisbane with a median titer of 60 (min <100, max 1200), and the response frequency in the non-adjuvanted group was 38% with a median titer of less than 100 (min <100, max 300).

**Figure 2:**
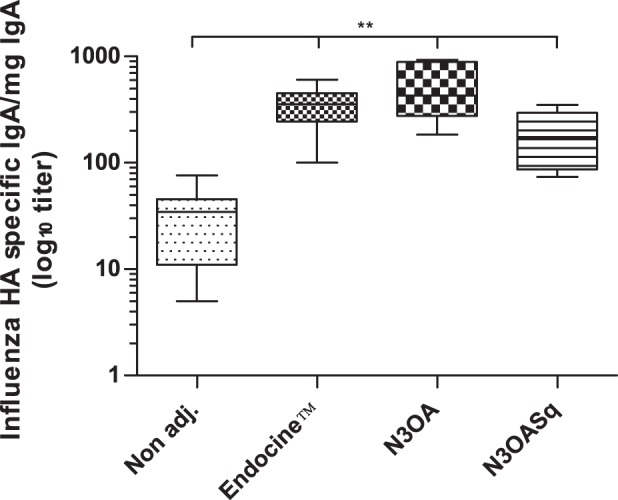
Mucosal influenza HA-specific IgA in nasal wash samples. Mice were immunized with a split influenza vaccine (Vaxigrip) containing the A/H1N1/Brisbane/2007 strain with or without adjuvant. The groups were immunized three times with three-week intervals. (A) Mucosal influenza HA-specific IgA in the nasal wash samples is shown. Median and range is shown for each group and statistical significance compared to the non-adjuvanted group is indicated, **p<0.003.

Nasal cavity wash fluids were analyzed after sacrifice; they showed that nasal IgA against influenza was significantly higher in all three adjuvanted groups compared to the non adjuvanted group (Fig. 2). The median influenza HA-specific IgA/mg total IgA titer in the Endocine™ group was 358, 427 in the N3OA group and the N3OASq group had a median titer of 175. The unadjuvanted group responded with a median nasal IgA titer of 35.

**Figure 3:**
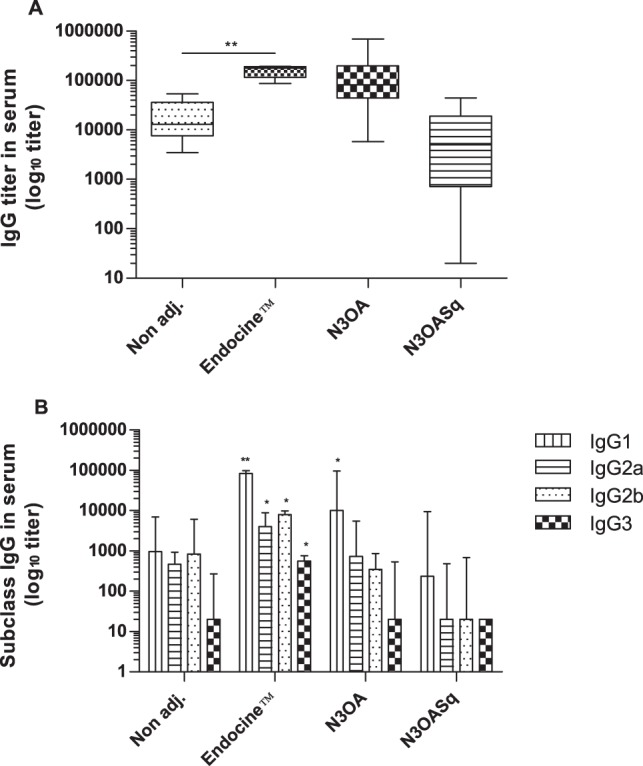
Humoral immune response in serum samples. Mice were immunized with a split influenza vaccine (Vaxigrip) containing the A/H1N1/Brisbane/2007 strain with or without adjuvant. The groups were immunized three times with three-week intervals. (A) A/Brisbane/59/2007 (H1N1) specific IgG ELISA titers in serum after the final immunization (B) A/Brisbane/59/2007 (H1N1) specific IgG subclass titers. Median and range is shown for each group. Values <100 is set as 20. Statistical significances compared to the non-adjuvanted group are indicated, *p<0.017, **p<0.003.

ELISA was used to determine the influenza-specific IgG response in the serum samples. The non-adjuvanted group of mice developed serum IgG titers against recombinant influenza A/Brisbane (H1N1) HA protein with a median titer of 13075. Significantly higher serum IgG titers were observed in the Endocine™ vaccinated group with a median titer of 179125 (Fig. 3A). Mice in the N3OA and N3OASq group developed IgG antibodies (median titer of 81640 and 4958) but not significantly higher than the non-adjuvanted group (Fig. 3A). Serum IgG against the rHA antigen of A/California/2009 (H1N1) was detected in the non-adjuvanted group with a median titer of 55170 (min 830, max 82500) and a median titer of 8385 (min 680, max 69500) in the Endocine™ group. The other two groups did not develop a detectable response (data not shown). Influenza A/Brisbane (H1N1) HA-specific serum IgG subclass titers were detectable in all eight mice in the Endocine™ vaccinated group. All animals in the Endocine™ group developed IgG1, IgG2a and IgG2b and 75% of the mice developed HA-specific serum IgG3 antibodies (Fig 3B). The non-adjuvanted group responded with mainly IgG1, IgG2a and IgG2b antibodies, the response frequency was 63–88%. In the N3OA group, the vaccinated mice responded with mainly IgG1 and IgG2a antibodies and 50% of the mice responded with IgG2b antibodies. The response frequency in the N3OASq group, when analyzing the IgG1, IgG2a and IgG2b response, was 25–50%. Only one mouse in the non-adjuvanted and the N3OA group developed IgG3 antibodies and none of the mice in the N3OASq group. Antibodies from mice in the non-adjuvanted and the Endocine™ vaccinated groups showed similar binding-strength or avidity toward the Brisbane virus HA, with an AI of 0.9–1.0. The N3OA and N3OASq groups showed similar avidity (AI of 0.8–0.9) and binding strength toward the A/H1N1/Brisbane strain (data not shown). Serum IgG antibodies against rHA of the two other strains in the vaccine given to the mice, H3N2 and B, could also be detected. High titers against influenza B was detected in the non-adjuvanted group, the Endocine™ group and in the N3OA group with median titers 543 200 (min 209700, max 4 060000), 163 200 (min 81900, max 683900) respectively 100 000 (min 3500, max 841400) (data not shown). There was no significant difference between these groups but comparing the non-adjuvanted group and the N3OASq group (median 2900) a significantly difference was seen in favor of the non-adjuvanted group. The antibody titers detected against the H3N2 strain were lower, but a significant difference could be detected when comparing the non-adjuvanted group median titer <100, (min <100, max 300) with the Endocine™ median titer 550, (min <100, max 8700) and N3OA group median titer 6300, (range min 900, max 9700) with p<0.0167and p<0.003 respectively (data not shown).

The ratio between IgG1 and IgG2a, a surrogate marker for TH2- or TH1-polarized immune responses respectively, suggests a dominant IgG1 (and thus TH2) immune response pattern with varying magnitude in all study groups. The median ratio between IgG1/IgG2a responses was 2.4 (min 0.3, max 49) in the non-adjuvanted group, 32.8 (min 9.9, max 82.3) in the Endocine™ group, 50.0 (min 13.3, max 85.3) in the N3OA group and 10.3 (min 1.0, max 38.8) in the N3OASq group (data not shown). The frequency of IgG2a responders were 63% in the non-adjuvanted group, 100% in the Endocine™ group, 86% in the N3OA group and lowest in the N3OA group with only 25% responders.

Serum IgA against rHA from influenza A/Brisbane (H1N1) was detectable in all animals in the Endocine™ vaccinated group with a median titer of 825. Compared to the non-adjuvanted group, were only 25% of the mice responded, the IgA response was significantly higher (p<0.003) in the Endocine™ group. In the cationic vaccinated groups only one animal per group responded with a serum IgA titer over 100 (Table 1). Serum IgA titers against rHA from the two other strains, influenza H3N2 and B, were also weak (data not shown). The response towards influenza B followed the same patterns as for H1N1. In the Endocine™ group all mice had detectable IgA titers against influenza B with a median titer of 628 (min 115, max 1450). Twenty-five percent of the mice in the N3OA group had detectable IgA titers but the median titer was <100. In both the non-adjuvanted group and the N3OASq group all mice had a titer <100. The IgA titers against the H3N2 strain was <100 in all mice in all four groups (data not shown). Serum IgA responses towards the split trivalent vaccine Vaxigrip, containing the Brisbane strains (H1N1, H3N2, B), were detected in all of the animals in the Endocine™ group with a median titer of 970 (Table 1). In the non-adjuvanted group, 88% of the animals responded with IgA antibodies to the Brisbane strains in Vaxigrip with a median titer of 665, but the difference between the non-adjuvanted group and the Endocine™ group was not significant. The mice immunized with the two cationic adjuvants responded with lower or no influenza-specific serum IgA, 43% of the animals in the N3OA group (median titer: <100) and only 13% of the animals in the N3OASq group (median titer: <100) had detectable influenza-specific IgA antibodies in serum. Serum IgA towards the split vaccine containing the A/California H1N1, A/Perth H3N2 and B/Brisbane strains was seen in 88% of the mice in the non-adjuvanted group, with a median titer of 980. All of the mice vaccinated with Endocine™ had a detectable influenza-specific IgA response, with a median titer of 860. The difference between the non-adjuvanted group and the Endocine™ group was not significant. The N3OA group responded poorly and only one mouse developed antibodies and none of the mice in the N3OASq group developed serum IgA antibodies against the split vaccine containing A/California H1N1, A/Perth H3N2 and B/Brisbane.

**Table 1:** Serum IgA titers against three different influenza stimuli (rHA, Vaxigrip and vaccine against influenza) after nasal vaccination. Median, range and response frequency in each group are shown.

Group	IgA titers against rHA (A/Brisbane (H1N1))	IgA titers against trivalent Vaxigrip (incl A/Brisbane (H1N1))	IgA titers against trivalent vaccine (incl A/California (H1N1))
**Non adjuvanted**	<100	665	980
	(<100–335, 25%)	(<100–7600, 88%)	(<100–10000, 88%)
**Endocine™**	825	970	860
	(100–970, 100%)	(750–2700, 100%)	(450–7800, 100%)
**N3OA**	<100	<100	<100
	(<100–680, 14%)	(<100–475, 43%)	(<100–100, 14%)
**N3OASq**	<100	<100	<100
	(<100–145, 13%)	(<100–145, 13%)	NA

Abbreviations: rHA =  recombinant hemagglutinin, NA  =  Not applicable.

### 3.2. Cell-mediated immune responses measured by ELISpot IFN-γ/IL-2

**Figure 4:**
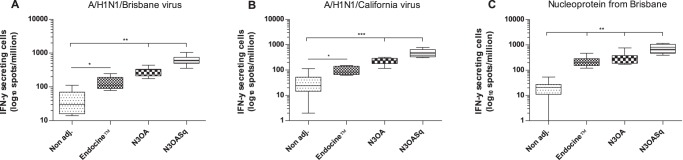
IFN-γ release from splenocytes, stimulated with different antigens. Mice were immunized with a split influenza vaccine (Vaxigrip) containing the A/H1N1/Brisbane/2007 strain with or without adjuvant. The groups were immunized three times with three-week intervals. Splenocytes were analyzed regarding IFN-γ release after influenza stimuli. (A) IFN-γ responses against influenza A/H1N1/Brisbane/59/2007 (whole virus). (B) IFN-γ responses against influenza A/H1N1/ California/04/2009 (whole virus). (C) IFN-γ responses against influenza nucleoprotein-peptides from A/H1N1/Brisbane. Median and range is shown for each group and statistical significance compared to the non-adjuvanted group are indicated, *p<0.017, **p<0.003 and ***p<0.0003.

The IFN-γ response (IFN-γ positive cells/million cells) against whole influenza virus (A/Brisbane/2007 (H1N1) and A/California/2009 (H1N1)) and against nucleoprotein (NP) CD8+ CTL epitope peptides (A/Brisbane (H1N1)) was measured in splenocytes collected after the final vaccination. The IFN-γ response was highest in the N3OASq group against all three antigens with a median number of 592 IFN-γ positive cells against A/H1N1/Brisbane/2007, a median number of 442 IFN-γ positive cells against A/H1N1/California/2009 and a median number of 671 IFN-γ positive cells against the NP peptides. The IFN-γ responses in the different study groups are shown in figure 4. All three adjuvanted groups significantly enhanced the influenza-specific IFN- γ release compared to the non-adjuvanted group.

**Figure 5:**
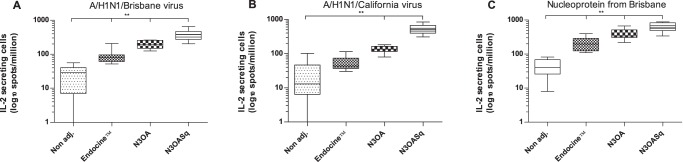
IL-2 release from splenocytes, stimulated with different antigens. Mice were immunized with a split influenza vaccine (Vaxigrip) containing the A/H1N1/Brisbane/2007 strain with or without adjuvant. The groups were immunized three times with three-week intervals. Splenocytes were analyzed regarding IL-2 release after influenza stimuli. (A) IL-2 responses against influenza A/H1N1/Brisbane/59/2007 (whole virus). (B) IL-2 responses against influenza A/H1N1/ California/04/2009 (whole virus). (C) IL-2 responses against influenza nucleoprotein-peptides from A/H1N1/Brisbane. Median and range is shown for each group and statistical significance compared to the non-adjuvanted group are indicated, *p<0.05, **p<0.01 and ***p<0, 001.

The IL-2 response (IL-2 positive spots/million cells) following the final booster was highest in splenocytes from the N3OASq group against the whole viruses A/Brisbane/2007 (H1N1) with a median number of 379 IL-2 positive cells, median number of 506 IL-2 positive cells against A/California/2009 (H1N1) and a median value of 614 IL-2 positive cells against the NP peptides. The IL-2 responses of the different study groups are shown in figure 5. As with the IFN-γ response, the N3OASq group had the strongest IL-2 response against all three influenza stimuli, but the N3OA and the Endocine™ group also responded with significantly higher IL-2 release than the non adjuvanted group. When splenocytes from the Endocine™ group were stimulated with A/California virus, a higher response could be seen compared to the non-adjuvanted group; however it was not significantly higher.

The strong IFN-γ and IL-2 responses against Brisbane and California whole virus originated mainly from CD4+ T cells, and a significant amount of cytokine production was lost when CD4+ T cells were depleted. The groups that had the highest ELISpot responses were the adjuvanted groups and they lost up to 90% of the IFN-γ secreting cells following CD4+ depletion (data not shown). Only the N3OASq group showed low but clearly detectable IFN-γ and IL-2 positive ELISpot populations after CD4+ T cell depletion *in vitro*, thus the IFN-γ and IL-2 positive reactivity remained in spite of CD4+ T cell depletion indicating the presence of a CD8+ T cells response. In conclusion, the N3OASq group had the strongest IFN-γ and IL-2 responses which were the product of both CD4+ and CD8+ T cells. The NP peptide mix was not tested on splenocytes after CD4+ T cell depletion.

## Discussion

In this study, three different mucosal adjuvants Endocine™, N3OA and N3OASq were evaluated together with a seasonal influenza vaccine containing A/H1N1/Brisbane/2007. The choice of adjuvant significantly affected the influenza A virus-specific humoral and cellular immune responses induced by intranasal vaccination in BALB/c mice.

The golden standard method to study the effect of influenza vaccines has been to use the HAI test. A HAI titer of 1∶32 or 1∶40 is considered to be a protective titer approximately in 50% of humans [27], [28], [29]. In this study a high frequency of mice developed HAI titers above 40 in two of the adjuvant (Endocine™ and N3OA) immunized groups. The HAI median titer was above 200 in groups vaccinated with vaccine containing Endocine™ or N3OA and the titers in these groups were significantly higher compared to the titers induced in mice given unadjuvanted vaccine where only 1/8 mice had a titer over 10. The same pattern was also seen with the NT-titers. The Endocine™ group and the N3OA group demonstrated significantly higher NT titers than the non-adjuvanted group. This shows that the vaccine and adjuvant is stimulating the humoral immune system to produce antibodies that can bind and inhibit the virus from infecting cells. By adding Endocine™ or N3OA to the split vaccine a significant increase in the neutralization antibody production is seen compared to the non-adjuvanted group. The N3OASq group responded with only two mice developing HAI antibodies, which means that these mice are not producing functional antibodies; they cannot bind and stop the virus from entering the host cell.

The mice in the three groups that were immunized with an adjuvant; Endocine™, N3OA or N3OASq, all had H1N1 influenza-specific mucosal IgA in the nasal cavity after the final vaccination. Secretory IgA has been shown to be able to protect against influenza infection in mice and to have cross- reactive properties in the upper respiratory tracts, while IgG protects the lower respiratory tracts and the lungs [10], [11], [30]. The first line of defense is the secretory-IgA antibodies in the mucosa, while the serum IgG antibodies work as the back-up antibodies. Secretory-IgA antibodies have also been shown to be more long-lived than the IgG antibodies in mucosa [31]. Endocine™ succeed to increase the production of influenza-specific serum IgG (H1N1 and H3N2), IgA (H1N1 and B strain) and mucosal IgA (H1N1) antibodies significantly compared to the non-adjuvanted group, while the cationic adjuvant N3OASq, only were able to stimulate a significantly higher production of mucosal IgA. The cationic adjuvant N3OA was able to stimulate a significantly higher production of H3N2-specific serum IgG and mucosal IgA towards the H1N1 strain.

The main focus in this study was the immune response against influenza A (H1N1) and the amount of antigen use for vaccination had previously been shown [19] to enhance the immune response towards H1N1 when administered together with Endocine™. In this study we used TIV and it was surprising to see that the serum response against the Influenza B/Brisbane/60/08 (WHO-Victoria-like influenza B) antigen was so strong also without any adjuvant. Previously studies have shown that there is cross-reactive antibody and CD4+ T cell responses between influenza B and A HAs in both humans and mice [32], [33], [34] and there is an estimated 23–27% amino acid homology between influenza B and A HA proteins [34], [35], [36]; which may be a reason for a strong IgG response towards influenza B. Also, in close proximity to the fusion peptide in the stalk of the HA, a highly conserved B cell epitope have been identified and shown to be cross-reactive within influenza A subtypes and influenza B HA [37]. Tamura et al have also suggested a broader immune recognition of antigens after mucosal immunization [35]. In a recent study by Skowronski et al 2011 were TIV:s with Influenza B/Brisbane/60/2008 (Victoria-like) were compared with the immunogenicity of influenza B/Florida/4/2006 (Yamagata-like) in BALB/c mice vaccinated parenterally, the immune responses against the first was relatively poor. In their study, the immune responses towards the Yamagata-like B strain were stronger than to the Victoria-like B strain. In our study, the serum IgG response to the influenza B/Brisbane was high even without a mucosal adjuvant. However, there are differences between our study and the work by Skowronski et al, most important in our study immunizations were performed nasally and in their study intramuscularly. Perhaps nasal administration with the B-strain is more immunogenic than parenteral administration, or the B antigen was more efficiently presented and was perhaps best promoted by cross-reactive epitopes induced both by the influenza A and B HAs and thereby creates a strong IgG response even without adjuvant. Interestingly, in the Skowronski study, also the A/H1N1/Brisbane/59/2009 strain was included. They demonstrated that the immunogenicity against the A/H1N1/Brisbane strain was relatively poor in contrast to the compared A/H1N1/California/7/2009 strain that also was included in the study [38]. Thus in this study, our nasal TIV vaccine candidate (with Endocine™ or N3OA) obtained fair humoral IgG immune responses towards all three influenza strains (A/H1N1/Brisbane/59/2007, A/H3N2/Brisbane/10/2007 and the B/Brisbane/60/2008) in comparison to what was obtained with the TIV given parentally. Further studies are needed to explain the obtained results, for instance by comparing the immunogenicity of each of the three influenza HAs, to perform more detailed epitope mapping and immunoglobulin avidity maturation after mucosal immunization in the mouse strain used.

The N3OA and N3OASq vaccinated mice failed to stimulate the production of H1N1 influenza-specific IgA in serum. Several studies [31], [39], [40] show that even if a high level of serum IgG is achieved and mucosal IgA is produced after intra nasal immunization, serum IgA is not produced or only produced at very low titers. Serum and mucosal antibodies are produced in different separated compartments in the body, secretory-IgA is produced by plasma cells in the nasal-associated lymphoid tissue (NALT) [40], [41], while serum antibodies are produced by plasma cells in the spleen and lymph nodes [42]. Thereby can different antibody titers be measured. However, it is likely that our nasal washes may contain both locally synthesized secretory-IgA and serum IgA since the murine mucosal system allow serum IgA to be transported to mucosal surfaces.

The two cytokines used to analyze the influenza-specific cellular response in this study, IL-2 and IFN-γ, are produced by T cells. IL-2 is an important cytokine for T cell activation and IFN-γ is a signature cytokine produced by cells mediating cellular immune responses directed against influenza virus infected cells [43]. To see if intranasal influenza vaccination had the capacity to induce influenza-specific IFN-γ and IL-2 responses, cellular responses were measured by ELISpot. Two of the peptides used in this experiment, NP_147–155_ and NP_141–155_, contain the CD8+ T cell epitope (TYQRTRALV) that is highly conserved between influenza strains [44] and has been used before in this kind of assay [45], [46]. The ELISpot assay showed that animals receiving the split vaccine together with the mucosal adjuvants had significantly stronger influenza-specific cellular responses on the day of sacrifice than mice receiving the non-adjuvanted vaccine formulation. This could be due to better delivery of the influenza antigens or enhanced immune stimulation in presence of the lipid based adjuvants. There is also a possibility that there is a difference in the kinetics of peak in the IFN-γ and IL-2 secretion of CD4+ T cells between non-adjuvant and adjuvant immunized animals. It was also seen that mice receiving the cationic adjuvant N3OASq produced significantly higher cytokine levels than the mice receiving the anionic-adjuvanted (Endocine™) vaccine formulation the only group that show CD8+ T cell epitope response. Cationic adjuvants have been described as having poor inflammation-inducing properties, but when combined with exogenous antigens, they can enhance the immune-activating properties of the chosen antigens. Cationic charges on liposomes can also provide better antigen-depot properties, which may favor cell-mediated immunity [47]. The size of the particles injected may also influence antigenic uptake, activation and presentation [48]. Nonionic, weakly anionic adjuvants and oil-in-water emulsions have been described as favoring the induction of TH2-type immunity [49], often through unresolved mechanisms. However, recent work [50], [51] has shown that injected oil-based emulsions can efficiently and rapidly recruit neutrophils, eosinophils and macrophages to the site of injection. These cells were shown to phagocytose injected adjuvant and deliver the antigen to draining lymph nodes, and the authors speculated that this could provide improved local antigen-presentation and enhanced immune response, especially humoral immune responses.

In this study we detected cross-reactive serum IgG antibodies against HA from A/California/2009 (H1N1) and serum IgA against a trivalent vaccine containing A/California/2009 (H1N1), in mice receiving the antigen alone and antigen together with Endocine™. When studying the splenocytes from the different immunization groups, significantly higher levels of IFN-γ and IL-2 could be detected after stimulation with both the A/California (H1N1) virus and the NP peptides in the adjuvanted groups compared to the non-adjuvanted group (the only exception being IL-2 production against California which was not significantly different between the Endocine™ group and the non-adjuvanted group). This confirms that nasal vaccine administration can generate a cross-reactive immune response. These T-cell results show similarities with results obtained with similar vaccines treated approximately the same way as in this study [52], [53], [54]. However, in the previous studies a ceramide-carbomyl-spermidine (CCS) cationic adjuvant was combined with influenza A trivalent vaccine given nasally.

Many influenza vaccine adjuvants intended for mucosal administration have been studied at the pre-clinical level [55], [56], [57]. However, only few mucosal adjuvant candidates have been approved and are safe enough to merit clinical studies [58]. The major hurdle is safety, especially in child vaccinations, when choosing the nasal route. One factor to consider is the close proximity to the olfactory nerves and the CNS when vaccinating intra-nasally. The advanced interest in using the Endocine™ [16] adjuvant arises from its components endogenous origin in human and animal cells. Endocine™ has been evaluated in three clinical studies in combination with vaccines against diphtheria, influenza and HIV and has shown a good safety profile in all studies. In the phase I/II influenza vaccination study, a special local tolerability study confirmed the tolerable profile of Endocine™ for nasal administration (manuscript in progress). The N3OA adjuvant has been evaluated pre-clinically and shown adjuvant effect when combined with DNA plasmids encoding HIV antigens [45]. The present study is the first study to evaluate the adjuvant N3OA with Sq (N3OASq). Other adjuvants like MF59 and AS03 also contain squalene and MF59 containing vaccines have been on the market for several years. AS03 is a newer adjuvant and was licensed in 2009 [59]. MF59 has shown good immune stimulating properties [51], [60], [61], but neither MF59 nor AS03 are approved for intra nasal vaccine administration.

In conclusion, we could see that both the Endocine™ adjuvant and the N3OA adjuvant together with influenza antigen induced significantly higher titers of influenza-specific mucosal IgA, HAI- and NT-antibodies compared to the non-adjuvanted group. All three adjuvants significantly increased the production of IFN-γ and IL-2, and thereby enhanced the cell-mediated immune response compared to the non-adjuvanted group. So, it was possible to induce both a stronger humoral and cell-mediated immune response when using Endocine™ and N3OA. Unfortunately we were not able to perform challenge studies, since mice were difficult to infect with the A/Brisbane/59/2007 (H1N1) strain (in-house experience, data not shown); otherwise this would have been of great interest. An earlier study by Petersson et al showed that mice nasally vaccinated with a subunit influenza vaccine in combination with 4% Endocine™ (previously denoted L3B), and that were challenged with A/New Caledonian (H1N1), had a significant lower copy number of viral RNA in the lungs compared to non-vaccinated mice [19]. Further analysis has to be done to investigate why the three adjuvants induced different immune responses. Different aspects possibly influencing the type of immune response induced could be the charge of the adjuvant, the different adjuvant compositions (which lipids that are included), the size of the adjuvant structure (liposomes), or how the antigen and adjuvant is gathered in the dispersion.
